# Bat coronaviruses related to SARS-CoV-2: what about their 3CL proteases (MPro)?

**DOI:** 10.1080/14756366.2022.2062336

**Published:** 2022-04-13

**Authors:** Matteo Pavan, Davide Bassani, Mattia Sturlese, Stefano Moro

**Affiliations:** Molecular Modeling Section (MMS), Department of Pharmaceutical and Pharmacological Sciences University of Padova, Padova, Italy

**Keywords:** SARS-CoV-2, bat coronavirus, sarbecovirus, BANAL, Laos, MPro, Paxlovid

## Abstract

Despite a huge effort by the scientific community to determine the animal reservoir of SARS-CoV-2, which led to the identification of several SARS-CoV-2-related viruses both in bats and in pangolins, the origin of SARS-CoV-2 is still not clear. Recently, Temmam et al. reported the discovery of bat coronaviruses with a high degree of genome similarity with SARS-CoV-2, especially concerning the RBDs of the S protein, which mediates the capability of such viruses to enter and therefore infect human cells through a hACE2-dependent pathway. These viruses, especially the one named BANAL-236, showed a higher affinity for the hACE2 compared to the original strain of SARS-CoV-2. In the present work, we analyse the similarities and differences between the 3CL protease (main protease, M^pro^) of these newly reported viruses and SARS-CoV-2, discussing their relevance relative to the efficacy of existing therapeutic approaches against COVID-19, particularly concerning the recently approved orally available Paxlovid, and the development of future ones.

## Introduction

Since its outbreak in December 2019, the COVID-19 pandemic has caused to date the death of almost 6 million people all around the world^1^^,^^2^. This worldwide-spread disease is caused by a betacoronavirus known as SARS-CoV-2, which infects the respiratory system of the host organism compromising its health status^3^. The symptoms of this illness range from the ones typical of influenza (cough, fever, and headache) to very serious complications such as breathing difficulty, pneumonia, and hypoxia, eventually leading to respiratory failure and death^4^. The high transmissibility of the SARS-CoV-2 virus allowed its fast diffusion all around the world, rapidly attracting the interest of experts in the medical, biological, and pharmaceutical environments, who have extensively worked and are still putting relevant efforts into the elaboration of proper solutions to fight this pathogen.

The first approach to finding viable therapeutic options was the so-called “drug-repurposing”, i.e. the use of drugs that are already marketed for the treatment of different diseases to cure COVID-19 patients. Concerning this, particular attention was directed towards HIV protease inhibitors such as Kaletra (therapeutic combination of Lopinavir and Ritonavir)^5^ and antimalarial drug Plaquenil (commercial name of hydroxychloroquine)^6^. Unfortunately, despite the promising premises (especially from a timescale perspective^7^), this approach was unsuccessful, with investigated drugs showing little to no efficacy in randomised clinical trials^8^.

Parallel to the first approach, a considerable amount of labour by both the industry and academia has been spent on developing tools that prevent the detrimental effect of the pathology and has resulted in the approval by the Food and Drug Administration (FDA) of several vaccines^9^. These therapeutic entities can be divided into three different classes^10^: the first one is composed of the inactivated virus vaccines, such as Chinese CoronaVac and the Russian CoviVac, the second family is formed by the ones based on adenovirus vectors, likeVaxzevria, Sputnik V, and the Janssen COVID-19 vaccine, while the third and final family consists of the mRNA-based ones such as the Pfizer-BioNTech “Comirnaty” and the Moderna “Spikevax”.

While vaccines based on inactivated viruses have given poor results, several studies have proven the efficacy of vaccination campaigns with the other two classes of vaccines (especially m-RNA ones) all around the world^11^^,^^12^. Despite the success of said vaccines, the SARS-CoV-2 Spike protein is often subjected to immune system-escaping mutations which lead to the development of new viral variants^13^, obliging the vaccines to be periodically updated to maintain their efficacy.

The high variability of the Spike protein among different coronavirus strains, which threatens the efficacy of already approved vaccines in the long period, led the scientific community to join forces to identify effective treatments for ongoing infections and to prevent future pandemic waves. Regarding this, a remarkable example is portrayed by the COVID Moonshot consortium, a collaborative project that involved scientists from all over the world in an attempt to design and develop an orally available drug against COVID-19^14^^,^^15^. COVID Moonshot aside, the great amount of knowledge accumulated on the target since the SARS-CoV epidemic in 2002/2003 rapidly resulted in the approval of the first COVID-19 specific treatments.

The first drug to be approved was Remdesivir, a polymerase inhibitor that was initially designed against the Ebola Virus and has then been repositioned against COVID-19. This drug, unfortunately, has an unfavourable pharmacokinetic profile, which limits its administration to the intravenous route in a hospital setting^16^^,^^17^. Tocilizumab, an interleukin-6 receptor monoclonal antibody originally developed to cure rheumatoid arthritis, obtained the emergency use authorisation (EUA) for the treatment of COVID-19 in the United States in June 2021^18^. The oral RNA-polymerase inhibitor Favipiravir has also been approved for marketing in countries such as Japan, China, India, Saudi Arabia, and the United Arab Emirates, but is still under examination from the FDA^19^.

An important milestone has been achieved at the end of 2021 with the FDA approval of the therapy based on the SARS-CoV-2 main protease (M^pro^) inhibitor Nirmatrelvir (also known as PF-07321332) in combination with Ritonavir, sold under the commercial name “Paxlovid” (which is available also in Europe since the end of January 2022)^20^. Thanks to its efficient, reversibly covalent inhibition of M^pro^, the Nirmatrelvir-based therapy demonstrated to lower of 89% the risk of severe complications after COVID-19 infection in symptomatic, unvaccinated, non-hospitalized adults^21^.

Recent scientific work by Temmam et al.reported the discovery of a high level of sequence similarity between the SARS-CoV-2 genome and that of other coronavirus species infecting cave bats living in North Laos^22^, raising serious concerns about the potential threat to public health that these coronaviruses could portray. Despite giving an in-depth analysis on the similarities and differences between the S protein of these newly reported viruses, no consideration is reported in the original work about their main proteases. For this reason, to assess the impact that these bat coronaviruses could have on public health, we performed a computational analysis to shed light on similarities and differences between the main protease of SARS-CoV-2 and that of these newly discovered bat coronaviruses, discussing the role that these alterations could have on the efficacy of existing therapies (Paxlovid, in particular) and the development of future ones.

## Materials and methods

The genome sequence for SARS-CoV-2, BANAL-52, BANAL-103, BANAL-236, and RaTG13 was obtained through GenBank. Table 1 reports the accession codes for each of the considered genomes. The protein sequence associated with the 3CL protease (main protease, M^pro^), was extracted, aligned using the appropriate tool from MOE 2019.01^23^, and used for the generation of the correspondent homology model (except for SARS-CoV-2, for which several crystal structures are available in the Protein Data Bank).

**Table 1.** The protein sequences used in this work and their origin.

Organism	Isolate	Accession Code	Product	Protein ID	Residues
SARS-CoV-2	“Wuhan-Hu-1”	NC_045512.2	ORF1ab polyprotein	YP_009724389.1	S3264-Q3569
Bat coronavirus	“BANAL-20-52/Laos/2020”	MZ937000.1	ORF1ab polyprotein	UAY13216.1	S3255-Q3560
Bat coronavirus	“BANAL-20-103/Laos/2020”	MZ937001.1	ORF1ab polyprotein	UAY13228.1	S3256-Q3561
Bat coronavirus	“BANAL-20-236/Laos/2020”	MZ937003.2	ORF1ab polyprotein	UAY13252.1	S3256-Q3561
Bat coronavirus RaTG13	“RaTG13”	MN996532.2	ORF1ab polyprotein	QHR63299.2	S3263-Q3568

The structure of SARS-CoV-2 main protease in its unliganded state was retrieved from the Protein Data Bank (PDB ID: 6Y2E^24^) and prepared using MOE 2019.01. At first, the functional dimer was restored applying the symmetric crystallographic transformation to each asymmetric unit. Secondly, residues with fractional occupancy values were assigned to the most probable state. Then, missing hydrogen atoms were added to the system according to the most probable protonation state at pH 7.4 for each titratable residue exploiting the “Protonate3D” tool. Afterward, hydrogen atoms coordinates were energy minimised according to the AMBER10: EHT force field until a gradient of 0.1 kcal mol^−1 ^Å ^− 2^ was reached. Finally, crystallographic water molecules were removed.

Four different homology models were generated exploiting the “Homology Model” tool, one for each bat coronavirus considered in the present work. The sequences used for the generation of homology models are reported in Table 1, while the structure 6Y2E, prepared as described before, was used as a template for the model generation.

## Results

**Figure 1.**
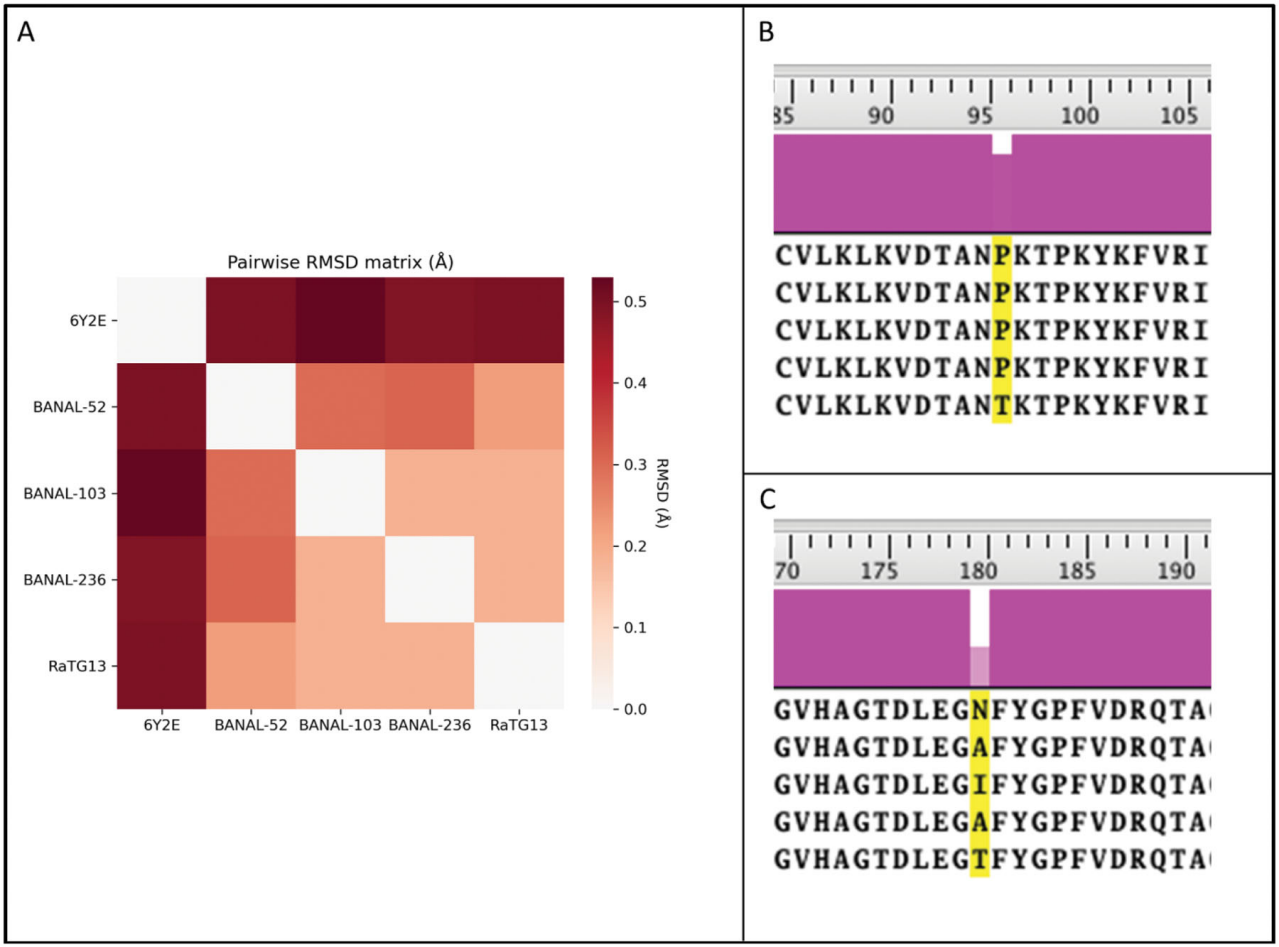
Comparison between SARS-CoV-2 3CL protease (M^pro^) from crystal structure 6Y2E (blue) and homology models of M^pro^ from four different bat coronaviruses, reported in Table 1. In Panel A, the pairwise RMSD matrix derived from the superposition of each bat coronavirus homology model to the template structure 6Y2E is reported. Panel B and C summarise the differences in the primary sequence between SARS-CoV-2 and bat coronaviruses M^pro^.

To compare similarities and dissimilarities between the SARS-CoV-2 M^pro^ and correspondent proteases in the most closely related bat coronaviruses, four different homology models (one for each different virus considered in this work) were generated, as reported in Materials and Methods. Due to the high degree of sequence identity (99,7% for BANAL-52, BANAL-103, and BANAL-236, 99,4% for RaTG13) between considered bat coronaviruses and SARS-CoV-2 M^pro^, homology modelling is expected to give a representative result, very closely related to the experimental data. As illustrated by Figure 1, there are only two differences in the primary sequences of considered viruses. These small changes to the amino acid sequences led to the generation of homology models that are practically superimposable to the reference structure (6Y2E), as is also depicted in Figure 1.

**Figure 2.**
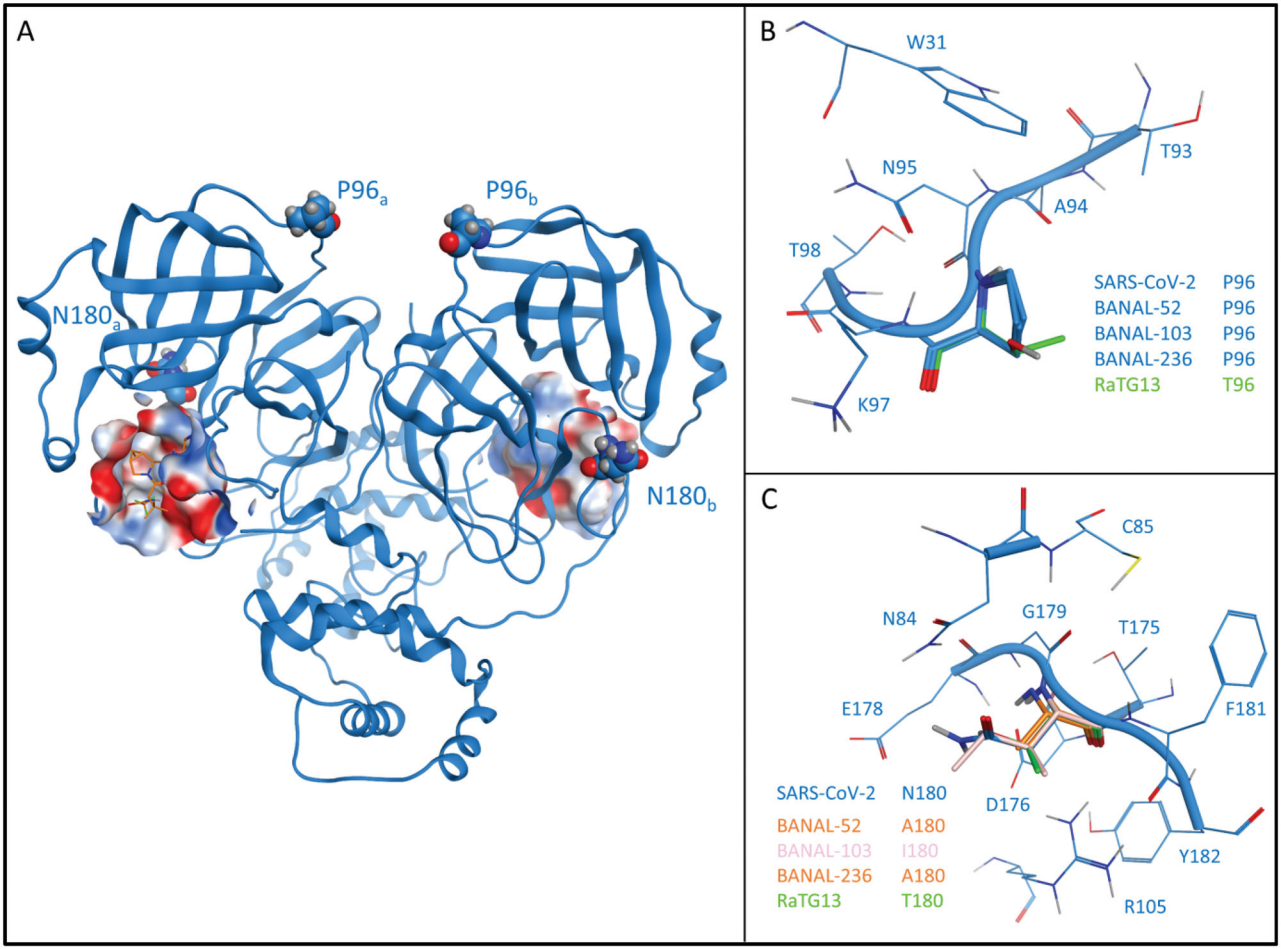
Comparison between SARS-CoV-2 3CL protease (M^pro^) from crystal structure 6Y2E (blue) and homology models of M^pro^ from four different bat coronaviruses, reported in Table 1. Panel A reports the structure of SARS-CoV-2 M^pro^ (PDB ID: 6Y2E) in its free form. The protein is depicted in blue ribbons, while mutated residues (namely, P96 and N180) in comparison with bat coronaviruses are highlighted and depicted as CPK models. For visual reference, Nirmatrelvir (also known as PF-07321332, commercial name Paxlovid) from structure 7RFS is also shown in the picture, alongside the binding site surface coloured according to electrostatic properties. Panel B highlights the comparison between residue 96 of SARS-CoV-2 M^pro^ and homology models of bat coronaviruses M^pro^. Panel C reports a comparison between residue 180 of SARS-CoV-2 M^pro^ and homology models of bat coronaviruses M^pro^.

Figure 2, instead, reports a comparison between the four homology models and SARS-CoV-2 M^pro^ from structure 6Y2E, mapping the differences between various proteases onto their three-dimensional structure.

The first difference is related to residue 96, which in the case of SARS-CoV-2 is a proline. This residue is conserved in each BANAL coronavirus reported by Temmam et al. but is not conserved in RaTG13, which was previously considered to be the most closely related bat coronavirus to SARS-CoV-2. Instead of a proline, RaTG13 presents a threonine residue at the 96 position, which is expected to increase both the flexibility and the hydrophilicity of the surroundings.

The second structural alteration is referred to residue 180, which in the case of SARS-CoV-2 is an asparagine. In this case, there is a higher variability between different coronavirus strains, with each BANAL virus presenting a hydrophobic residue (alanine, in the case of BANAL-52 and BANAL-236, isoleucine in the case of BANAL-103), while RaTG13 once again differentiate from both BANAL viruses and SARS-CoV-2 presenting a hydrophilic threonine residue.

## Discussion

The comparison between the crystal structure of SARS-CoV-2 and homology models of bat coronaviruses M^pro^showed that there are two main structural differences, both of which do not involve the catalytic site.

In the native SARS-CoV-2 structure, Phe96 is involved in a series of hydrophobic contacts with the side chain of Trp31, Thr93, and Lys97 through its pyrrolidine core. In the case of RaTG13, the only bat coronavirus that presents an alteration at this position, the presence of a threonine causes a reduction of possible hydrophobic contacts with the surrounding amino acids but does not cause the loss of any crucial interaction for structural integrity. Moreover, this residue is located in a solvent-exposed flexible loop region that connects between two beta-sheets, a further indication that this substitution should not compromise the structural integrity of the protease.

Concerning the second structural alteration, in the native SARS-CoV-2 structure Asn180 is involved in a double interaction with the sidechain of two charged residues, namely Asp176 and Arg105. Both of these interactions happen with the backbone of Asn180 and do not involve its sidechain, which is stretched towards the solvent. Intriguingly, in this case, the newly discovered bat coronaviruses all present a hydrophobic residue at position 180: in all these cases, no loss of native interaction happens, coherently with the fact that they do not involve the sidechain of residue 180 and only occur through its backbone. Once again, RaTG13 is the most diverse one, being the only analysed bat coronavirus that presents a polar amino acid (a threonine) at this position. As previously mentioned, the sidechain of residue 180 is not involved in any structurally relevant interaction, and therefore the presence of a hydroxyethyl sidechain does not give a particular advantage to this virus strain. Furthermore, as is the case for Pro96, this structural modification is also located in a solvent-exposed, non-structured loop region, indicating that no critical harm to the protease integrity should be provoked by this alteration.

Altogether, our structural analysis reveals that neither of these two structural differences between SARS-CoV-2 and bat coronaviruses M^pro^ should determine any relevant structural alteration of the main protease. Notably, this observation is in agreement with a recent article that characterised the effect of each possible M^pro^ mutation on its functionality: both Pro96 and Asn180 are marked as highly tolerant to mutations^25^.

Concerning the implications of these two mutations on the efficacy of M^pro^ inhibitors, several elements point to the conclusion that neither mutation should have a relevant effect. As can be seen from Figure 2, which gives a depiction of the localisation of these two mutations relative to the position of the catalytic site (which is also the binding site of most protease inhibitors, including PF-07321332, the active principle of Paxlovid) shows that both these mutations are not directly linked to the catalytic site, indicating that the binding cleft that harbours PF-07321332 should not be altered. Moreover, as thoroughly assessed in previous scientific work from our laboratory, neither of these two residues is in any way involved in the recognition process of PF-07321332^26^, complementing the structural information provided by crystal structures 7VH8^27^, 7RFS, and 7RFW which clearly show how none of this two residues contributes to the interaction with PF-07321332 in the final bound state.

The fact that the SARS-CoV-2 main protease and the one from closely related bat coronaviruses are very similar and practically identical at the catalytic site supports the idea that targeting this protease is still a viable therapeutic option not only for the present but also for the prevention of future pandemic waves.

To date, several studies have contributed to thoroughly characterising the nature of the shallow and solvent-exposed catalytic site of the SARS-CoV-2^28^, which has proven to be readily investigable with both time-dependent and time-independent structure based-approaches such as molecular docking^29^ and molecular dynamics^30^, leading to the development of compounds with affinities in the low nanomolar range^31^^,^^32^.

All these factors, combined with the fact that striking 3 D structure similarities exists also with other coronaviral M^pro^ such as the one from Porcine transmissible Gastroenteritis virus (TGEV)^33^, Human coronavirus strain 229E (HCoV)^34^, Infectious bronchitis virus (IBV)^24^and MERS-CoV^35^, validate the pursue of novel M^pro^ inhibitors that could increase the pool of available treatment for COVID-19 and also for future coronavirus-related diseases, acting as pan-coronaviral drugs.

## Conclusions

Recently, a scientific work by Temmam et al. reported the discovery of bat coronaviruses closely related to SARS-CoV-2 that can infect human cells. This scientific work raised the attention of both the scientific community and the general audience to the possible threat to public health that these newly discovered coronaviruses could represent. Despite a thorough characterisation of Spike protein of these bat coronaviruses, no information was given in the original work about their main proteases, which is considered the main target for the development of COVID-19 specific active principles.

In the present scientific work, we performed a computational analysis to characterise structural similarities and differences between the main proteases of SARS-CoV-2 and closely related bat coronaviruses. A comparison between the crystal structure of SARS-CoV-2 M^pro^ and homology models of bat coronavirus M^pro^ shows that two main differences exist, involving the mutation of Pro96 and Asn180. None of these structural alterations are predicted to have an impact on the protease structural integrity, functionality, or affinity for existing inhibitors (especially the recently approved orally available Paxlovid), nor towards the development of novel protease inhibitors. Furthermore, the high degree of structural conservation among main proteases from different coronaviruses suggests that M^pro^ is not only a valid target for the treatment of COVID-19, but that the knowledge acquired on this target could be useful in the identification and development of pan-coronaviral drugs that can cure different diseases and prevent future pandemic waves.

## References

[1] Guarner J. Three emerging coronaviruses in two decades: the story of SARS, MERS, and now COVID-19. Am J Clin Pathol 2020;153:420–1. 32053148 10.1093/ajcp/aqaa029 PMC7109697

[2] COVID Live - Coronavirus Statistics - Worldometer. Available from: www.worldometers.info/coronavirus/ [last accessed 04 Apr 2022].

[3] Zhou F, Yu T, Du R, et al. Clinical course and risk factors for mortality of adult inpatients with COVID-19 in Wuhan, China: a retrospective cohort study. Lancet 2020;395:1054–62. 32171076 10.1016/S0140-6736(20)30566-3 PMC7270627

[4] Centers for Disease Control and Prevention, ‘Interim Clinical Guidance for Management of Patients with Confirmed Coronavirus Disease (COVID-19)’. Available from: www.cdc.gov/coronavirus/2019-ncov/hcp/clinical-guidance-management-patients.html [last accessed 04 Apr 2022].

[5] Bolcato G, Bissaro M, Pavan M, et al. Targeting the coronavirus SARS-CoV-2: computational insights into the mechanism of action of the protease inhibitors lopinavir, ritonavir and nelfinavir. Sci Rep 2020;10:20927. 33262359 10.1038/s41598-020-77700-z PMC7708625

[6] Gautret P, Lagier J-C, Parola P, et al. Hydroxychloroquine and azithromycin as a treatment of COVID-19: results of an open-label non-randomized clinical trial. Int J Antimicrob Agents 2020;56:105949. 32205204 10.1016/j.ijantimicag.2020.105949 PMC7102549

[7] Mani D, Wadhwani A, Krishnamurthy PT. Drug repurposing in antiviral research: a current scenario. J Young Pharm 2019;11:117–21.

[8] Viveiros Rosa SG, Santos WC. Clinical trials on drug repositioning for COVID-19 treatment. Rev Panam Salud Publica 2020;44:e40. 32256547 10.26633/RPSP.2020.40 PMC7105280

[9] Fiolet T, Kherabi Y, MacDonald CJ, et al. Comparing COVID-19 vaccines for their characteristics, efficacy and effectiveness against SARS-CoV-2 and variants of concern: a narrative review. Clin Microbiol Infect 2022;28:202–21. 34715347 10.1016/j.cmi.2021.10.005 PMC8548286

[10] Pollard AJ, Bijker EM. A guide to vaccinology: from basic principles to new developments. Nat Rev Immunol 2021;21:83–100. 33353987 10.1038/s41577-020-00479-7 PMC7754704

[11] Moghadas SM, Vilches TN, Zhang K, et al. The impact of vaccination on coronavirus disease 2019 (COVID-19) outbreaks in the United States. Clin Infect Dis 2021;73:2257–64. 33515252 10.1093/cid/ciab079 PMC7929033

[12] Rinott E, Youngster I, Lewis YE. Reduction in COVID-19 patients requiring mechanical ventilation following implementation of a national COVID-19 vaccination program - Israel, December 2020-February 2021. MMWR Morb Mortal Wkly Rep 2021;70:326–8. 33661863 10.15585/mmwr.mm7009e3 PMC7948930

[13] Harvey WT, Carabelli AM, Jackson B, et al. SARS-CoV-2 variants, spike mutations and immune escape. Nat Rev Microbiol 2021;19:409–24. 34075212 10.1038/s41579-021-00573-0 PMC8167834

[14] Consortium TCM, et al. Open science discovery of oral non-covalent SARS-CoV-2 main protease inhibitor therapeutics. *bioRxiv* 2020.10.29.339317 2022

[15] Morris A, McCorkindale W, Consortium TCM, et al. Discovery of SARS-CoV-2 main protease inhibitors using a synthesis-directed de novo design model. Chem Commun 2021;57:5909–12. 10.1039/d1cc00050k PMC8204246 34008627

[16] Kokic G, Hillen HS, Tegunov D, et al. Mechanism of SARS-CoV-2 polymerase stalling by remdesivir. Nat Commun 2021;12:1–7. 33436624 10.1038/s41467-020-20542-0 PMC7804290

[17] Beigel JH, Tomashek KM, Dodd LE, et al. Remdesivir for the treatment of Covid-19 — final report. N Engl J Med 2020;383:1813–26. 32445440 10.1056/NEJMoa2007764 PMC7262788

[18] Xu X, Han M, Li T, et al. Effective treatment of severe COVID-19 patients with tocilizumab. Proc Natl Acad Sci U S A 2020;117:10970–5. 32350134 10.1073/pnas.2005615117 PMC7245089

[19] Manabe T, Kambayashi D, Akatsu H, Kudo K. Favipiravir for the treatment of patients with COVID-19: a systematic review and meta-analysis. BMC Infect Dis 2021;21:1–13. 34044777 10.1186/s12879-021-06164-x PMC8159019

[20] Owen DR, Allerton CMN, Anderson AS, et al. An oral SARS-CoV-2 M pro inhibitor clinical candidate for the treatment of COVID-19. Science 2021;374:1586–93. 34726479 10.1126/science.abl4784

[21] Hammond J, Leister-Tebbe H, Gardner A, et al. Oral nirmatrelvir for high-risk, nonhospitalized adults with Covid-19. N Engl J Med 2022. 10.1056/NEJMoa2118542 PMC8908851 35172054

[22] Temmam S, Vongphayloth K, Baquero E, et al. Bat coronaviruses related to SARS-CoV-2 and infectious for human cells. Nature 2022. 10.1038/s41586-022-04532-4 35172323

[23] Molecular Operating Environment (MOE), 2019.01; Chemical Computing Group ULC, 1010 Sherbooke St. West, Suite #910, Montreal, QC, Canada, H3A 2R7, 2021. Available from: https://www.chemcomp.com/Research-Citing_MOE.htm.

[24] Xue X, Yang H, Shen W, et al. Production of authentic SARS-CoV M(pro) with enhanced activity: application as a novel tag-cleavage endopeptidase for protein overproduction. J Mol Biol 2007;366:965–75. 17189639 10.1016/j.jmb.2006.11.073 PMC7094453

[25] Flynn JM, Samant N, Schneider-Nachum G, et al. Comprehensive fitness landscape of SARS-CoV-2 M^pro^ reveals insights into viral resistance mechanisms. bioRxiv 2022.01.26.477860. 2022. 10.7554/eLife.77433 PMC9323007 35723575

[26] Pavan M, Bolcato G, Bassani D, et al. Supervised molecular dynamics (SuMD) insights into the mechanism of action of SARS-CoV-2 main protease inhibitor PF-07321332. J Enzyme Inhib Med Chem 2021;36:1646–50. 34289752 10.1080/14756366.2021.1954919 PMC8300928

[27] Zhao Y, Fang C, Zhang Q, et al. Crystal structure of SARS-CoV-2 main protease in complex with protease inhibitor PF-07321332. Protein Cell 2021:1–5. 10.1007/s13238-021-00883-2 PMC8533666 34687004

[28] Fornasier E, Macchia ML, Giachin G, et al. A new inactive conformation of SARS-CoV-2 main protease. Acta Crystallogr. Sect. D Struct. Biol 2022;78:363–78. 35234150 10.1107/S2059798322000948 PMC8900819

[29] Bassani D, Pavan M, Bolcato G, et al. Re-exploring the ability of common docking programs to correctly reproduce the binding modes of non-covalent inhibitors of SARS-CoV-2 protease Mpro. Pharmaceuticals 2022;15:180. 35215293 10.3390/ph15020180 PMC8878732

[30] Bissaro M, Bolcato G, Pavan M, et al. Inspecting the mechanism of fragment hit binding on SARS‐CoV‐2 Mpro by using supervised molecular dynamics (SuMD) simulations. ChemMedChem 2021;16:2075–81. 33797868 10.1002/cmdc.202100156 PMC8250706

[31] Zhang C-H, Stone EA, Deshmukh M, et al. Potent noncovalent inhibitors of the main protease of SARS-CoV-2 from molecular sculpting of the drug perampanel guided by free energy perturbation calculations. ACS Cent Sci 2021;7:467–75. 33786375 10.1021/acscentsci.1c00039 PMC7931627

[32] Luttens A, Gullberg H, Abdurakhmanov E, et al. Ultralarge virtual screening identifies SARS-CoV-2 main protease inhibitors with broad-spectrum activity against coronaviruses. J Am Chem Soc 2022;144:2905–20. 35142215 10.1021/jacs.1c08402 PMC8848513

[33] Anand K, Palm GJ, Mesters JR, et al. Structure of coronavirus main proteinase reveals combination of a chymotrypsin fold with an extra alpha-helical domain. EMBO J 2002;21:3213–24. 12093723 10.1093/emboj/cdf327 PMC126080

[34] Anand K, Ziebuhr J, Wadhwani P, et al. Coronavirus main proteinase (3CLpro) structure: basis for design of anti-SARS drugs. Science 2003;300:1763–7. 12746549 10.1126/science.1085658

[35] Ho B-L, Cheng S-C, Shi L, et al. Critical assessment of the important residues involved in the dimerization and catalysis of MERS coronavirus main protease. PLoS One 2015;10:e0144865. 26658006 10.1371/journal.pone.0144865 PMC4682845

